# Distress and quality of life characteristics associated with seeking surgical treatment for stress urinary incontinence

**DOI:** 10.1186/1477-7525-7-8

**Published:** 2009-02-05

**Authors:** Karen M Gil, Amber M Somerville, Sara Cichowski, Jennifer L Savitski

**Affiliations:** 1 Department of Obstetrics and Gynecology, Akron General Medical Center, Akron, OH, USA; 2 Northeastern Ohio Universities College of Medicine and Pharmacy, Rootstown, OH, USA

## Abstract

**Background:**

Current research focuses on three variables in evaluating the impact of stress urinary incontinence (SUI) on daily living: severity of incontinence, distress or bother resulting from incontinence, and effect on health related quality of life (HRQoL). Understanding the impact of these variables is important as they are the driving force behind women seeking surgical treatment. Given the importance of HRQoL in determining need for treatment, as well as evaluating treatment success, this review provides an assessment of the degree to which HRQoL is impaired in women seeking surgical treatment.

**Methods:**

PubMed searches for the terms "quality of life and distress and urinary incontinence" and "quality of life and bother and urinary incontinence" were performed with limits of English, human and female subjects through May 2008. All studies using validated instruments were included. No time limit was placed on the search.

**Results:**

Of 178 articles retrieved, 21 met the inclusion criteria, and 17 reported methods of scoring. The studies used the Urogenital Distress Inventory (UDI) and the Incontinence Impact Questionnaire (IIQ). Wide ranges of mean and individual levels of severity of symptoms, UDI and IIQ scores were seen among women seeking surgical treatment. Fourteen studies reported baseline and post-surgical treatment distress and QoL data. Statistically significant improvements between baseline and post-surgical UDI and IIQ scores were reported in 12 studies. Reported cure rates ranged from 46% to 97%. Satisfaction with the procedure was reported in 4 studies and ranged from 84% to 91%. A minority of studies reported the relationship between reduction in symptoms and change in HRQoL.

**Conclusion:**

HRQoL is the main reason women seek surgical treatment for incontinence and surgical treatment leads to a significant improvement in mean HRQoL scores. Assessment of HRQoL has proved less useful in identifying why individual women seek treatment for incontinence. Preliminary work has begun to characterize the interaction between severity of symptoms, distress or bother resulting from these urinary symptoms, impact on HRQoL, and treatment seeking behavior, but further research is needed. Greater standardization in the reporting of results of distress or bother and HRQoL would allow for comparison across studies.

## 

Urinary incontinence (UI) is defined by the International Continence Society as the "complaint of any involuntary leakage of urine" [1]. Urinary incontinence is associated with significant reductions in health related quality of life (HRQoL) [2-8]. Prevalence estimates of UI among community dwelling women range from 10 to 40%, while estimates for institutionalized women are greater than 50% [9]. The wide range in estimates stem, in part, from differences in definitions of UI and characteristics of the population studied [10].

Stress UI (SUI) is an involuntary leakage of urine with effort or exertion, sneezing or coughing [1]. Among incontinent women, estimates are that 50% suffer from SUI [11]. Treatment for SUI is primarily surgical and is largely patient driven. There are a variety of measures of severity of UI, but none has emerged as the gold standard [12]. In the absence of a clear-cut standard for providing incontinence surgery that is based on severity, indication for treatment is based on the extent a woman's HRQoL is affected. HRQoL is traditionally a concept that has been used to "assess the patient's overall sense of well-being and how it relates to the disease and the disease treatment" [13]. Patient reported outcomes (PRO) are often viewed as a secondary endpoints in order to measure symptom control and treatment toxicities in conditions such as gynecologic cancer [14] where the primary outcome is generally survival. In comparison, PRO may be the primary end point in seeking treatment for SUI and the primary end point in assessing treatment effectiveness.

Recent studies have examined the role of distress or bother in assessing the impact of UI on HRQoL in community dwelling women, in part to begin to determine what factors affect treatment seeking behavior [2-6]. In general, these studies found that women who experienced greater symptom bother or distress reported a greater impact on HRQoL, however the majority do not seek treatment [2,4-6]. It is not known what factors ultimately lead some women to seek treatment for UI and then undergo surgery. A recent pan-European study of 9487 women who had sought treatment for UI found that severity of symptoms was the most important predictor of QoL and bother [8]. Assessment of severity of symptoms, degree of distress or bother, and impact on QoL has not been systematically investigated in women who have elected to undergo surgery. A review of these variables will provide a framework for further investigating why some women elect not to seek treatment, and will provide an informational base to assess treatment success using PRO. This study investigates the level of distress or bother, and impact on QoL, resulting from SUI in women who have elected to undergo surgery.

## Methods

Two searches were conducted using the PubMed version of Medline. The first search used the terms "quality of life and distress and urinary incontinence" and the second used the terms "quality of life and bother and urinary incontinence." The searches were limited to studies published in English conducted on human, female subjects. The search was conducted through May 2008 and no time limit was placed on the search. Validation or testing of outcome measures and prevalence studies were not included. Articles were excluded if subjects were not incontinent, if women were receiving treatment for a medically necessary condition such as bladder cancer or if data from women with SUI were not presented separately from women with other forms of UI.

All articles using validated instruments, in their entirety (not just a few questions) that reported scores for the whole instrument were included. All validated instruments were included. The Symptom and Quality of Life Committee of the International Consultation on Incontinence performed a systematic review of questionnaires related to urinary and anal incontinence between 2001 and 2004 [15]. They identified 23 robust and relevant questionnaires, including 14 Grade A questionnaires relevant to the assessment of urinary incontinence in women. Instruments that combined symptoms and QoL impact were the International Consultation on Incontinence Questionnaire (ICIQ) [16], the Bristol Female Lower Urinary Tract Symptoms Questionnaire-Short Form (BFLUTS-SF) [17], and the Stress and Urge Incontinence and Quality of Life Questionnaire (SUIQQ) [18]. Instruments that addressed urinary incontinence symptoms were the Urogenital Distress Inventory (UDI) [19] and the UDI-6 [20], the Incontinence Severity Index (ISS) [21] and the BFLUTS [22]. Instruments that examined QoL impact of urinary incontinence were the Incontinence QoL questionnaire (I-QoL) [23]), The Stress-related leak, Emptying ability, Anatomy, Protection, Inhibition, Quality of life, Mobility and Mental status Incontinence Classification System (SEAPI-QMM) [24], the King's Health Questionnaire (KHQ) [25], the Incontinence Impact Questionnaire (IIQ) [19], the IIQ-7 [20], the Urinary Incontinence Severity Score (UISS) [26] and the CONTILIFE: a Quality of Life questionnaire for urinary incontinence [27]. Non-validated instruments that were excluded from this review mainly consisted of asking patients to what degree they felt bothered by their symptoms and to what degree their symptoms impacted their quality of life on a Likert type scale.

All assessments of severity were included. Assessments of severity included questionnaires such as the Urinary Incontinence Severity Scale (UISS) [26] and the Sandvik Incontinence Severity Index (ISS) [28]. A validated questionnaire, adapted from the Medical, Epidemiological and Social Aspects of Aging (MESA) survey includes questions specifically designed to assess the frequency of stress related urine loss [29]. Measures of severity often include information obtained from a voiding diary or from patients' recall of the number of episodes of incontinence or leaks or the number of pads used during a time period [12,30-32]. Objective measurements, such as long or short term measurements of change in pad weight, bladder emptying by measuring residual volume, and urodynamic measures are also included in patients' evaluation [12,30,31,33]. Urodynamic testing to evaluate urethral function, bladder capacity and stability is often recommended before surgery for SUI [31].

All definitions of cure rate were included, and are based on definitions of stress urinary incontinence made during the evaluation. Definitions of cure therefore include no reports of incontinence, and no loss of urine during urodynamic testing, a cough test, or pad testing. Patient satisfaction has been measured with a visual analog scale (VAS), and a Patient Satisfaction Questionnaire (PSQ) that has not been validated, but includes a question asking patients how satisfied they are with their progress (completely, somewhat, or not at all satisfied) [34].

## Results

### Search

**Figure 1:**
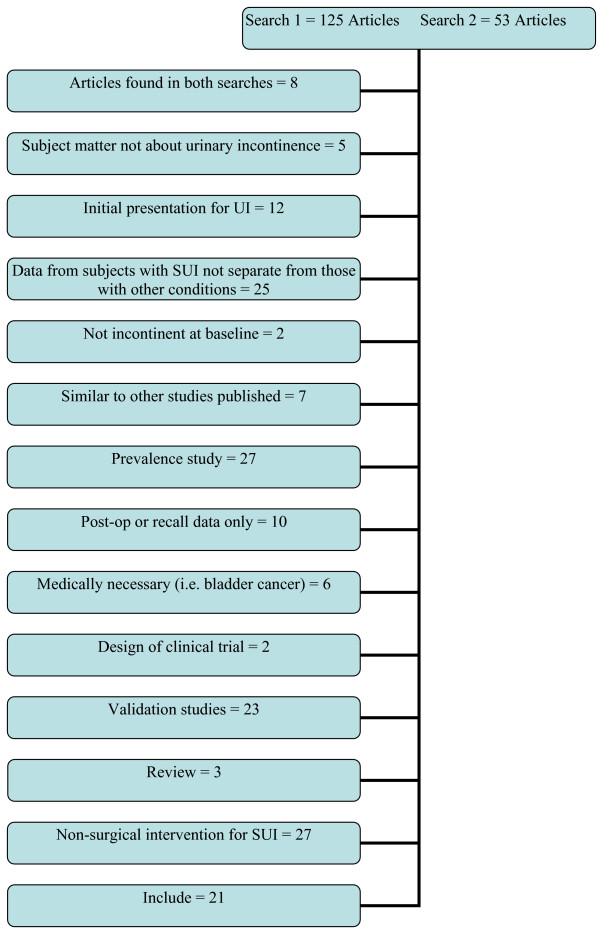
**Results of search 1 (quality of life and distress and urinary incontinence) and search 2 (quality of life and bother and urinary incontinence)**.

Using the search strategy described in the methods section, 178 articles were identified. The articles were independently reviewed by two of the authors to determine if they met inclusion criteria. Articles were then independently reviewed by the other two authors for verification and 21 articles met criteria. Information regarding the articles that did not meet the criteria is reported in Figure 1 in accordance with meta-analyses of observational studies (MOOSE) guidelines. The only quality criteria used for inclusion was use of a validated instrument for measurement of QoL; strength of the study design is included in Table 1 [see Additional file 1].

The majority of the studies were designed to test the effectiveness of surgical treatments for stress urinary incontinence. Several surgical options exist to treat stress urinary incontinence. The choice of procedure depends on several factors including surgical risk of the patient, skill of the surgeon and coexisting prolapse/pathology. The Burch procedure is a retropubic operation that can be performed via the open or laparoscopic approach. The Burch procedure attempts to restore the normal anatomic position of the proximal urethra and bladder neck. The endopelvic fascia of the mid and proximal urethra is attached to Cooper's ligament located on the posterior aspect of the superior pubic ramus. In contrast, sling procedures act to support the bladder neck and urethra providing anatomic stability and a base for urethral compression during episodes of increased abdominal pressure. The sling can be autologous or non-autologous fascia or synthetic mesh. The sling is placed beneath the proximal or mid-urethra, depending on procedure type, from a vaginal incision with the ends either brought retropubically to the anterior abdominal wall (proximal suburethral fascial sling, tension-free vaginal tape (TVT)) or brought laterally to the groin (transobturator tape (TOT)). The sling procedures require only a small vaginal incision to isolate the proximal or mid urethra, and two small exit sites for the ends of the sling, thus making the sling procedures considerably less invasive than the Burch procedure.

While all validated instruments were eligible, almost all of the studies reported results using either the short or long forms of the Urogenital Distress Inventory (UDI) and the Incontinence Impact Questionnaire (IIQ). The UDI is a questionnaire that assesses bother or distress related to symptoms of UI [19]. The long form of the UDI consists of 19 questions and total scores are converted to a score range of 0–300, with higher scores representing greater symptom distress. A short form, the UDI-6, consists of six questions that are representative of the long form [20]. Scores are converted to a possible range of 0–100. The IIQ is a questionnaire that measures the impact of UI on physical activity, social relationships, travel, and emotional health [19]. The long form consists of 30 questions and scores are converted to a possible range of 0–400 with higher score representing greater impact on QoL. A revised long form, the IIQ-R, was developed that also takes into account symptom embarrassment [35]. A short form, the IIQ-7, consists of seven questions which are representative of the long form and scores are converted to a possible range of 0–100 [20]. Four studies that met criteria and used these instruments were not included since the scoring used was not clear and an assessment of the degree of bother and impact on QoL could not be determined without knowing the maximum possible score [36-39], leaving 17 studies for review [40-56] (Table 1) [see Additional file 1]. Three studies presented baseline data using standardized QoL instruments among women presenting for surgical treatment but no post-treatment data or only change data (no absolute values) and 14 studies presented data before and after surgery

### Pre-operative Data

Severity was assessed with a questionnaire in three studies [41,42,46]; mean values were within the third and fourth quartile. Urine diaries were used in three studies [42,51,55] and pad weight measurements were used in three studies [42,45,49]; large ranges in severity were recorded, including reports of no incontinence episodes over the testing period [42,55] or no change during a pad weight test [42,49].

With one exception, all studies mean or median values for the UDI long or short form were very close to the midpoint of the scale; the range in mean or median scores around the midpoint were 38% [53] to 67% [54], but most studies reported mean values close to 50% of the possible score. One study reported mean scores that were only 14% of the maximum possible score [51]. Several studies reported the range of scores and in some cases, the range of scores women reported included 0 or values close to 0 [42-44,53]. In two studies, the lowest reported scores were at the 42–44 percentiles [45,50]. There was a great deal of variability in the highest reported score; some studies reported the highest values that were close to the maximum [42-44], while others reported lower maximum values [45,50,53].

Reported mean IIQ scores for either the long or short form were also near the midpoint of the scale with mean scores ranging from 33% [53] to 65% [52]. Many studies that reported the range of IIQ scores also reported scores that were 0 or close to 0 [42-44,53]; in two studies, the lowest reported IIQ score was 33% [50] or 43% of the maximum [45]. The maximum value in studies reporting ranges of IIQ scores were close to the maximum in some [42-44,53], but not all studies [45,50].

### Post-Operative Data

Large decreases in severity measurements were noted in the studies measuring severity after surgical treatment (Table 1) [see Additional file 1].

Statistically significant improvements between baseline and post-treatment UDI and IIQ scores were reported in 12 studies, including those that only reported change values [40,41,44,45,47-51,54-56]. No studies reported that changes in mean or median scores were not significantly different. Mean or median UDI scores post-operatively for all reported studies fell within, or close to, the first quartile. Mean or median IIQ scores post-operatively were either 0 or close to 0, or fell within the first quartile.

Ten studies reported cure rates from the surgical interventions. Reported cure rates varied from a low of 46% [46] to a high of 96.8% [44,51]. Assessments used varied widely from not requiring further surgery for incontinence, to a negative cough stress test. Four studies reported patient satisfaction with the surgery with satisfaction scores ranging from 84 to 91%.

### Relationships Between Measures

Several studies examined the relationship between severity of incontinence and measures of distress or bother and QoL. One study specifically examined whether the bladder volume at which urodynamic stress incontinence is first detected is related to scores on the UDI and IIQ [43]. Baseline median IIQ and UDI scores did not differ significantly by volume group [43]. An additional study also found no correlation between urodynamic measures of stress incontinence and either UDI or IIQ scores, but there was a weak to moderate positive relationship between number of reported leaks per day and UDI and IIQ scores (UDI r = 0.23; IIQ r = 0.34) and a moderate relationship between patient report of the frequency of stress related urine loss (MESA score) and UDI and IIQ scores (UDI r = 0.46; IIQ r = 0.47) [42]. A high correlation between 1 hour pad test results and UDI and IIQ scores was reported in one study (UDI r = 0.40, p < .001; IIQ r = 0.69, p < .001) [45]. Murphy et al. examined the relationship between ISI (Sandvik) scores and the UDI and IIQ [46]. The ISI was significantly correlated with the UDI (r = 0.36, p < .001) but was not significantly correlated with the IIQ (r = 0.27, p = 0.08). Hagen et al. used regression modeling to demonstrate that greater urine leakage and episodes of incontinence were significantly associated with increased UDI and IIQ scores [55].

Examining symptoms remaining after surgery and UDI and IIQ scores, Bakas et al. examined changes in UDI and IIQ scores in patients who were cured, improved or unchanged (cure was defined as change in one hour pad weight of less than 1 g; improvement was reduction in urine loss to less than 50% of urine loss measured pre-operatively) [45]. UDI and IIQ scores decreased significantly in cured patients (median UDI scores decreased from 45 to 20; IIQ scores decreased from 48 to 0). UDI scores decreased to a lesser extent in women who were improved (48 to 23, p = 0.03) and were not significantly different in women who were unchanged (49 to 30, p = 0.06); IIQ scores were not significantly decreased in women who were improved (63 to 47, p = 0.09) or unchanged (55 to 56, p = 0.31). Domingo et al. reported that patients who presented with SUI symptoms post-operatively (10% of the patients), did not demonstrate a significant change in mean UDI or IIQ scores [44]. Murphy et al. found highly significant correlations between post-operative ISI (Sandvik) scores and the UDI (r = 0.78, p < 001) and IIQ (p = 0.70, p < .001) [46]. Hagen et al. found a highly significant positive relationship between decrease in number of incontinence episodes and total UDI and IIQ scores [55].

Relationships between UDI and IIQ were examined at baseline and post-operatively. One study reported a moderate correlation between UDI and IIQ at baseline (r = 0.58) [42]. A related study using the same population of patients examined the relationship between clinical and demographic factors associated with IIQ scores [57]. They found that lower IIQ scores are associated with greater symptom bother (standardized beta coefficient 0.39, p < .001).

## Discussion

PRO have proved crucial in identifying treatment success for urinary incontinence, and surgical treatment results in significant improvements in mean HRQoL scores (Table 1). Comparison of outcomes using novel approaches, such as tension free vaginal tape (TV) and transobturator tape (TOT), often in comparison to the "gold standard" of Burch colposuspension, routinely use the UDI and IIQ to assess relative effectiveness [40,42,43,51,53].

PRO have proved less useful in identifying why women seek treatment for incontinence. The range in UDI and IIQ scores among women who sought treatment is large (Table 1). The majority of studies that included ranges reported women at both extremes – some women reported no distress or impact on activities of daily living while others reported close to the maximum level. Severity of symptoms also appears to be of limited usefulness in assessing why women seek surgical treatment. Some women sought treatment despite reporting incontinence episodes occurring as infrequently 0 times during a 3 day period [42] or per 48 hours [55].

A clear cut relationship between measures of severity and measures of distress, bother and impact on HRQoL pre-operatively has not been determined. Several studies specifically examined the relationship between severity of incontinence and UDI and IIQ scores, and while generally positive [42,45,46,55] the correlations were often not very strong [42,46]. Urodynamic measures of severity have even less consistent results, with two studies reporting no correlation with UDI and IIQ scores [42,43]. A recent study used linear regression analysis to examine clinical measures of severity, including urodynamic measurements, as predictors of QoL, measured with the UISS, and found greater leakage in a 48 hour pad test was a significant, yet modest, predictor of decreased QoL (beta 0.25, p = 0.034) [26]. It has been suggested that the underlying dissociation between severity measures and QoL is due to the subjective perception of symptoms by the individual or a difference in the tolerance of symptoms by individual patients [15]. Objective measures of severity do not account for variations in lifestyle that affect the subjective experience of the patient [5].

Similar to the baseline data, post treatment UDI and IIQ scores demonstrated wide ranges both within study groups and between studies (Table 1). Several studies have begun to examine the effect of improvement versus resolution of symptoms on changes in HRQoL measurements, and have found a positive relationship between change in symptoms and improvement in PRO [44-46,55]. Stach-Lempinen et al. found that the change in urine leakage measured with a 48 hour pad test best, but modestly, predicted the change in QoL scores, measured with the UISS, in a linear regression analysis, (beta 0.30, p = 0.024) [26]. Fitzgerald et al. also specifically examined changes in UDI and IIQ score following incontinence surgery and found the post-operative UDI and IIQ scores were higher among women with stress incontinence symptoms than among women without them [39]. More extensive exploration of changes in severity of incontinence and changes in HRQoL would provide information on how patients perceive the success of a surgery that does not result in complete elimination of symptoms.

Surgical intervention is not without risks, and the available procedures may not result in a complete resolution of symptoms, [Table 1, 58,59]. There is no clear consensus on how to evaluate success following surgery for SUI [58,59]. A recent review of treatment effectiveness found that only seven out of 37 randomized clinical trials used validated questionnaires to assess symptoms of UI and their impact on QoL, but seven different instruments were used making comparisons impossible [58]. They also found that there was no standardization to the assessment of severity, so that outcomes could not be compared across studies.

Treatment of incontinence is different from other fields of health care in that HRQoL is seen as the central goal, not as a secondary outcome. It is women's subjective experience of their SUI symptoms that drive them to seek treatment and as such, treatment is focused on improving HRQoL rather than survival. However, women are presenting for surgery with a wide range of objective and subjective assessments of severity of symptoms and impact on daily life. Some studies have begun to report on the relationship between women's perception of the distress or bother resulting from SUI and the impact that it has on the quality of their life, however further research should explore the extent to which SUI symptoms causes distress and the extent to which this distress impacts daily life. A more complete understanding of why women are presenting for surgery will then aid in the identification of related outcome measures to use. The relationship between resolution of symptoms, improvement in HRQoL and overall satisfaction with the procedure may provide information that will enable women who currently have SUI to have a better understanding of the risks and benefits of surgery. For this to be achieved, standardized measurements using the same scoring procedures should be used to allow for comparison between various interventions and studies.

## Conclusion

HRQoL appears to be affected by the degree to which women are bothered by SUI symptoms. This may contribute to whether a woman chooses to seek treatment for her SUI. Distress or bother and QoL are both improved following surgical intervention for SUI. Further studies are needed to fully explain the relationship between distress or bother and QoL, and greater standardization in the reporting of results of distress or bother and QoL measures will allow for comparison among studies.

## Abbreviations

HRQoL: Health related quality of life; IIQ: Incontinence Impact Questionnaire; ISI: Incontinence Severity Index (Sandvik); PRO: Patient Reported Outcomes; PSQ: Patient Satisfaction Questionnaire; SUI: Stress Urinary Incontinence; TVT: Tension Free Vaginal Tape; UDI: Urogenital Distress Inventory; UISS: Urinary Incontinence Severity Score; TOT: Transobturator Tape; VAS: Visual Analog Scale

## Competing interests

The authors declare that they have no competing interests.

## Authors' contributions

Two of the authors (KG and AS) made substantial contributions to the conception and design of the study, acquisition of data, data analysis and interpretation of data. All authors contributed to data analysis, interpretation of the data, writing of the manuscript and revised it critically for important intellectual content. All authors have participated sufficiently in the work to take public responsibility for the content of the paper.

## Supplementary Material

Additional file 1 **Table 1.** Pre- and post-operative data from women receiving surgical treatment for stress urinary incontinence. Pre- and post-operative quality of life data from women who have elected to undergo surgical treatment for stress urinary incontinence are provided. Click here for file
