# Supplementary material for: Distress and quality of life characteristics associated with seeking surgical treatment for stress urinary incontinence
Source: Health Qual Life Outcomes. 2009 Feb 5;7:8. doi: 10.1186/1477-7525-7-8 (PMC2645374; doi:10.1186/1477-7525-7-8)
Supplement: Additional file 1 — Table 1. Pre- and post-operative data from women receiving surgical treatment for stress urinary incontinence. Pre- and post-operative quality of life data from women who have elected to undergo surgical treatment for stress urinary incontinence are provided. [file 1477-7525-7-8-S1.doc]

| Reference | Severity  Measurement  (possible  range) Baseline Post-Surgery | | | Urogenital Distress Inventory  Version  (possible Baseline Post-Surgery  range) (range) (range) | | | Incontinence Impact Questionnaire  Version  (possible Baseline Post- Surgery  range) (range) (range) | | | Measure: Success rate | Satisfaction |
| --- | --- | --- | --- | --- | --- | --- | --- | --- | --- | --- | --- |
| Murphy, USA [40]*, Ambidirectional cohort study of women with SUI receiving TVT (n=97) or TOT (n=232) |  |  |  | UDI-6  (0-100) | TVT:  56.6 + 18.3^a^  TOT:  49.4 + 20.2^a^ |  | IIQ-7  (0-100) | TVT:  43.2 + 25.6^a^  TOT:  38.9 + 26.3^a^ |  |  |  |
| Sung, USA, [41]* Retrospective chart review of women with SUI,  <60 years old (n=168);  >60 years old (n=81) | ISI Sandvik Score  (0-12) | <60 years old:  6.8+0.5^a^  >60 years old:  7.4+0.8^a^ |  | UDI-6  (0-100) | <60:  48.3 + 1.8^a^  >60:  45.8 + 2.6^a^ |  | IIQ-7  (0-100) | <60:  43.5 + 2.2^a^  >60:  34.6 + 3.1^a^ |  |  |  |
| Albo, USA [42]** Multi-center, randomized clinical trial of Burch colposuspension compared to pubovaginal sling in women with stress predominant symptoms (SISTEr Trial) (n=655) | MESA,  ( 0-45)  Leaks/day, 3-day voiding diary  24 hr pad wt (gm) | 26 + 7 ^a^  (4-43)  3.2 + 3.0^a^  (0-26)  43.5 + 79.4^a^  (.1 – 1022) |  | UDI  (0-300) | 151 + 49^a^  (0-291) |  | IIQ  (0-400) | 171 + 101^a^ (0-400) |  |  |  |
| Lowenstein, USA  [43]. Retrospective chart review of consecutive women with SUI undergoing rectus fascial sling or Burch colposuspension (n=168). Follow-up: 3 months |  |  |  | UDI-6  (0-100) | 56^b^  (0-100) | Median values ranged from 11 to 17 (range 0-83) (groups based on initial urodynamic SI volume) | IIQ-7  (0-100) | 38^b^ (0-100) | Median values ranged from 0 to 5 (range 0-76) (groups based on initial urodynamic SI volume) | Did not have persistent USI: 73% |  |
| Domingo, Spain [44] Retrospective chart review of women with SUI or MUI undergoing TOT (n=93). Follow-up: 1 year. |  |  |  | UDI-6  (0-100) | 49 + 21^a^ (3-100) | 15 + 15^a^ (0-66) | IIQ-7  (0-100) | 40 + 28^a^  (0-100) | 9 + 15^a^  (0-76) | Cough stress test  96.8% |  |
| Bakas, Greece [45] Prospective evaluation of women with SUI undergoing TVT (n=89). Follow-up: 12 months (range 6-18 months) | 1 hour pad test (gm) | 51.5 + 28.6g^a^  (7-147) | 1.5 + 2.5g^a^  (0-20) | UDI-6  (0-100) | 45^c^ (42-47) | 20 (18-21)^c^ | IIQ-7  (0-100) | 49 (43-56)^c^ | 0 (0-0)^c^ | Pad weight  < 1 g:  87.6% |  |
| Murphy, USA [46] Prospective evaluation of women with SUI undergoing TVT (n=137). Follow-up: 32 months (range 18-51 months) | ISI Sandvik Score  (0-12) | 9.8 + 2.8^a^ | 3.7^d^ | UDI-6  (0-100) | 59.8 + 19.3^a^ | 26.5^d^ | IIQ-7  (0-100) | 47.8 + 28.4^a^ | 17.8^d^ | Sandvik score = 0:  46.1% |  |
| Schraffordt Koops, Netherlands  [47] Prospective, multicenter, cohort study of women with SUI undergoing TVT (n=634). Follow-up: 2 months |  |  |  | UDI-6  (0-100) | 58.0 + 0.8^e^ | 25 + 0.9^e^ | IIQ-7  (0-100) | 58.0 + 0.8^e^ | 15 + 1.1^e^ | “Do you leak during physical activity, coughing or sneezing”  (% improved):  92% |  |
| Richter, USA [48]  Prospective evaluation of women with SUI undergoing TVT (n=73). Follow-up: 1 month |  |  |  | UDI-6  (0-100) | 61.8 + 19.9^a^ | 21.9 + 19.2^b^ | IIQ-7  (0-100) | 51.1 + 29.6^a^ | 12.7 + 23.5^a^ | Negative cough stress test:  91.2% | PSQ: 91.2% somewhat or completely satisfied |
| Tomoe, Japan,  [49] Prospective evaluation of women with SUI undergoing TVT (n=66). Follow-up: 2 years | 1 hour pad test (g/hr) | 26.8 ^d^ (range: 0-175) |  | UDI-6  (0-100) | 40^d^ | 5^d^ | IIQ-7  (0-100) | 45^d^ | 3^d^ |  | 88% were satisfied or much satisfied |
| Segal, USA  [50] Retrospective chart review of women with SUI undergoing TVT (n=98). Follow-up: 3 months |  |  |  | UDI-6  (0-100) | 56^f^  (44-71) | 17 ^f^  (6-28) | IIQ-7  (0-100) | 52 ^f^  (33-71) | 0 ^f^  (0-5) | Did not need additional anti-continence procedure: 95.9% |  |
| Paraiso, USA  [51] Prospective randomized clinical trial to compare the laparoscopic Burch colposuspension (LBC)  (n= 33) with the TVT (n=33) in women with SUI. Follow-up: 21 months (range 12-43 months) | Incon-tinence episodes/wk (one week urine diaries) | LBC:  16 + 13^a^  TVT:  16 + 14^a^ | LBC:  0.4 + 1.6^a^  TVT:  1.8 + 5.1^a^ | UDI  (0-300) | LBC:  40 + 14^a^  TVT:  42 + 15^a^ | LBC:  4 + 2^a^  TVT:  6 + 2^a^ | IIQ  (0-400) | LBC:  144 + 90^a^  TVT:  164 + 95^a^ | LBC:  38 + 51^a^  TVT:  49 + 38^a^ | Did not have urodynamic SI  LBC:  81.2%  TVT:  96.8% | VAS, 0 = not at all satisfied to 10 = extremely satisfied  LBC:  8.4 + 2.9  TVT:  8.5 + 2.8 |
| Crivellaro, USA  [52]. Prospective evaluation of women with SUI undergoing transvaginal sling procedure  (n=234). Follow-up: 9 months | Pads/day (patient report) | 3.0 ^d^ | .5 ^d^ | UDI-6  (0-18) | 11.6^d^ | 3.1^d^ | IIQ-7  (0-21) | 13.7^d^ | 2.7^d^ | 78% were dry or improved (slight leak) |  |
| Hung, Taiwan  [53]. Prospective evaluation of consecutive women with SUI undergoing a pubovaginal sling (PVS) (n=57) or TVT (n=23). Mean follow-up: 20 (PVS) and 23 (TVT) months. |  |  |  | UDI-6  (0-100) | TVT:  49.3^d^  (0-72.2)  PVS:  38.3^d^  (0-66.7) | 11.6^d^  (0-44.4)  9.3^d^  (0-38.9) | IIQ-7  (0-100) | TVT:  39.7^d^  (0-95.2)  PVS:  33.1^d^  (0-95.8) | TVT:  6.7^d^  (0-38.1)  5.7^d^  (0-37.5) | Negative cough stress test or no reports of leaking during stress:  TVT:  65.2%  PVS:  71.9% |  |
| Richter, USA  [54] Prospective evaluation of women with SUI undergoing cadaveric fascia sling procedure. Follow-up: 12 months |  |  |  | UDI-6  (0-100) | 67.1 + 24.2^a^  (n=83) | 28.0 + 22.9^a^  (n=76) | IIQ-7  (0-100) | 55.1 + 32.4^a^  (n=82) | 11.0 + 19.5^a^  (n=76) |  | PSQ: 90.2% somewhat or completely satisfied  (n=82) |
| Hagen, Scotland  [55] Prospective evaluation of women seeking SUI surgical intervention. Follow-up: 3 months | Incon-  tinence episodes/ 48 hrs (48 hour urine diaries) | 4^b^  (0-23)  (n=83) | 0^b^  (0-3)  (n=69) | UDI  (0-300) | 140.0 + 55.8^a^  (n=83) | 27.8 + 37.7^a^  (n=69) | IIQ  (0-400) | 174.2 + 10.2^a^  (n=83) | 40.5 + 8.1^a^  (n=69) |  |  |
| Vassallo, USA  [56] Prospective evaluation of women with SUI undergoing TVT (n=116). Mean follow-up: 22 months (range 6-50 months) |  |  |  | UDI-6  (0-100) | 50.4^d^ | 20.8^d^ | IIQ-7  (0-100) | 41.3 ^d^ | 9.5 ^d^ |  |  |

*Post-operative data were presented as change values and are not included. ** Only pre-operative data presented. ^a^Mean + SD; ^b^Median (Range); ^c^Median (95% CI); ^d^Mean; ^e^Mean + SEM; ^f^Median (Interquartile Range),
